# Preparation of cationic proteoliposomes using cell-free membrane protein synthesis: the chaperoning effect of cationic liposomes†

**DOI:** 10.1039/d0ra05825d

**Published:** 2020-08-04

**Authors:** Mitsuru Ando, Yoshihiro Sasaki, Kazunari Akiyoshi

**Affiliations:** Department of Polymer Chemistry, Graduate School of Engineering, Kyoto University Katsura, Nishikyo-ku Kyoto 615-8510 Japan akiyoshi@bio.polym.kyoto-u.ac.jp +81-75-383-2590 +81-75-383-2589

## Abstract

Membrane protein reconstituted cationic liposomes are constructed using cell-free membrane protein synthesis in the presence of cationic liposomes. The chaperon effect of cationic liposomal membrane assists in folding the functional conformation of membrane protein. This preparation method enables the provision of the usage of proteoliposomes for drug delivery.

## Abstract 2

The preparation method of cationic proteoliposomes is established using a cell-free membrane protein synthesis in the presence of cationic liposomes.

## Graphical abstract

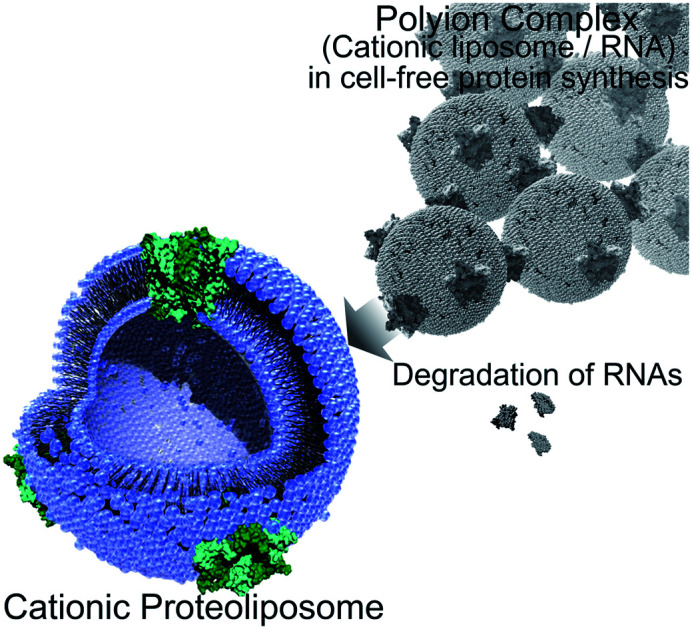

Membrane proteins have been increasingly studied for their use in advanced applications in the drug delivery system (DDS) field, including for membrane protein-conducted drug delivery^1^ and for delivery of the membrane protein itself into the plasma membrane.^2^ Membrane proteins carry out their unique biological activities with high specificity toward their particular substrate. Specific cell strains show cell type-dependent membrane protein expression, which may include certain proteins clustered together.^3^ In addition, a portion of cell-type specific intercellular communication is orchestrated by membrane proteins through peer-to-peer interactions such as a gap junction, desmosome, immune check point, and antigen presenting major histocompatibility complex-T cell receptor.^4^ Thus, the functions of membrane proteins are attractive for use in the development of advanced drug delivery systems as new devices in nanomedicine. Although proteoliposomes, membrane proteins reconstituted into liposomes, have been used for these purposes, it remains difficult to express membrane proteins using a cell-based procedure. Often the hydrophobic nature of membrane proteins often hampers their isolation with a bioactive conformation. Therefore, in DDS, membrane proteins are mostly used for developing natural lipid carriers such as cell-derived lipid vesicles^1*a*,2*b*^ or for incorporation into a viral envelope.^5^

Contrary to cell-based protein preparation, cell-free protein synthesis^6^ can occur in the presence of detergents,^7^ lipidic scaffolds^8^ or cytotoxic compounds.^9^ Therefore, cell-free protein synthesis is useful for screening medicines for binding to a target molecule^10^ and for preparing solubilized membrane proteins.^11^ Previously we developed a method to prepare proteoliposomes using cell-free membrane protein synthesis in the presence of liposomes (*i.e.*, a cell-free membrane protein synthesis/liposome system).^12^ In this procedure, the chaperoning effect of the liposomal membrane prevented aggregation of the hydrophobic proteins and assisted in integrating the synthesized nascent membrane proteins into the liposomal membrane to form bioactive membrane proteins and oligomers.^12,13^ Using connexin-43 (Cx43) proteoliposomes prepared by this procedure, we demonstrated the cytosolic delivery of small molecules through the Cx gap junction to cells.^14^ However, the preparation of proteoliposomes still suffers from low yields of synthesized protein and insufficient incorporation efficiency of some membrane proteins.^15^

The targets of cargo medicines are often located in the subcellular compartment. For the intracellular delivery of their cargoes, DDS nanocarriers have to attach to and be internalized across the negatively charged plasma membrane and then escape from the endosomes. In general, cationic nanocarriers such as cationic liposomes electrostatically interact with the negatively charged plasma membrane, and then are internalized through several endocytosis pathways. The cationic properties of cationic nanocarriers induce the proton sponge effect in endosomes leading to their osmotic rupture, and the release of cargo medicines into the subcellular compartment.^16^ One type of cationic lipid, 1,2-dioleoyl-*sn*-glycero-3-ethylphosphocholine chloride salt (DOEPC), is widely used to provide the positive charges on the liposomal surface.^17^ DOEPC liposomes allow the lipids to mix with the negatively charged phospholipid membrane leading to the efficient escape of the DOEPC liposomes and their cargoes from the endosomes.^18^

In this study, we report the preparation of cationic proteoliposomes using DOEPC and a cell-free membrane protein synthesis/liposome system and describe the chaperoning effect of these cationic liposomes, in which Cx43 was selected as a model membrane protein.

To evaluate the effect of the positive charge of the liposomes on cell-free membrane protein synthesis, we prepared cationic liposomes using varying amounts of DOEPC as the cationic lipid. The average diameter and zeta potentials of each liposome are summarized (Table S1, ESI†). The average diameters of each liposome were between 125 nm and 145 nm. The zeta potentials of the liposomes increased in a DOEPC concentration-dependent manner. The cell-free Cx43 synthesis was performed in the absence or presence of various cationic liposomes (0.5 mM lipid) for 4 h at 37 °C in a heat block incubator (Fig. 1A). After the cell-free protein synthesis, the amount of Cx43 synthesized was evaluated by western blot analysis using an antiCx43 antibody. The amount of synthesized cell-free Cx43 was similar in the absence (100 ± 21.2) % and presence of neutral DOPC liposomes (100%). In the presence of 1 mol% and 2 mol% DOEPC liposomes, the protein synthesis efficiency slightly decreased, from (88.2 ± 11.6) % for 1 mol% DOEPC liposomes to (86.2 ± 11.1) % for 2 mol% DOEPC liposomes compared with protein synthesis in the presence of neutral DOPC liposomes. In contrast, when DOEPC was 4 mol%, the cationic liposomes inhibited the cell-free Cx43 synthesis as shown by the yield of (70.1 ± 17.6) %. At 8 mol% and 16 mol%, the DOEPC liposomes strongly inhibited the cell-free Cx43 synthesis as the amounts of Cx43 synthesized were (48.9 ± 12.1) % and (15.5 ± 4.04) %, respectively, compared with protein synthesis in the presence of DOPC liposomes.

**Fig. 1:**
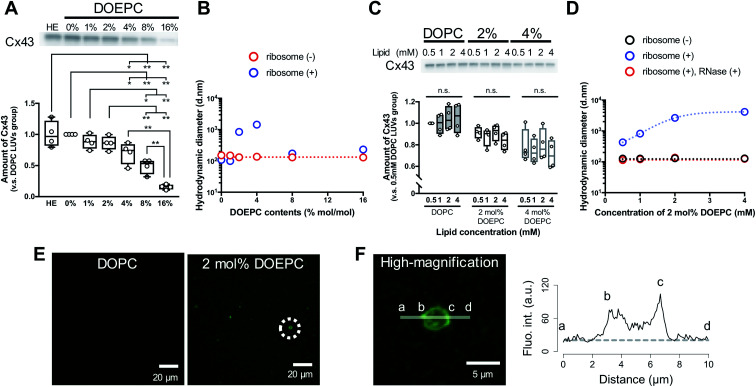
Polyion complex of cationic liposomes with nucleic acid components in cell-free protein synthesis. (A) western blot analysis of cell-free Cx43 synthesized in the absence or presence of cationic liposomes. Each dot denotes an individual data point (*n* = 4). The line denotes the mean. (B) Average hydrodynamic diameter by intensity using dynamic light scattering with or without incubation with liposomes and ribosomes. (C) western blot analysis of cell-free Cx43 synthesized in the presence of various concentrations of cationic liposomes. Each dot denotes an individual data point (*n* = 4). The line denotes the mean. (D) Average hydrodynamic diameter by intensity using dynamic light scattering with or without incubation with liposomes and ribosomes. After incubation, ribosomes were degraded by mixing with an RNase. (E) Confocal laser scanning microscopy image of cell-free reactants after cell-free Cx43 synthesis in the presence of each liposome. Green signals represent SYBR Gold. (F) Re-captured high-magnification image of an object inside a dash circle in (E) and the fluorescence intensity profile of SYBR Gold on the indicated line. The dash line represents the background level of the fluorescence intensity.

To understand why cationic liposomes inhibited the cell-free membrane protein synthesis, we focused on the interaction of the cationic liposomes with anionic ribosomes, which are one of the main components in a cell-free reaction mixture. Interaction of cationic liposomes and ribosomes in 50 mM HEPES buffer was evaluated by dynamic light scattering (DLS) (Fig. 1B). The size of the *E. coli* ribosome used in this study was 23.1 nm (PdI: 0.066) measured by DLS. After the addition of ribosomes to the DOPC liposomes or 1 mol% DOEPC liposomes, the average sizes did not change much. By comparison, when the ribosomes were incubated with 2 mol% and 4 mol% DOEPC liposomes, the average sizes dramatically increased to 845 nm and 1444 nm, respectively. However, in the presence of 8 mol% and 16 mol% DOEPC liposomes, the average sizes were similar to the value in the absence of ribosomes. This liposomal and ribosomal behavior is similar to the polyion complex formation by cationic and anionic polyelectrolytes.^19^

The cationic DOPEC content-dependent decrease of Cx43 production may be attributed to the electrostatic interactions between the cationic liposomes and other anionic nucleic acid components of the reaction mixture such as the tRNAs and plasmid DNAs in addition to the ribosomes. These electrostatic interactions might inhibit mRNA transcription from pDNA and in turn, protein production. According to the central dogma of molecular biology, transcription is regulated by epigenetic modification of histones.^20^ The acetylation of lysine residues in histones decreases their positive charges,^21^ which leads to attenuation of their electrostatic interactions with DNA and transcription of coded genes. Thus, disruption of electrostatic interactions by cationic liposomes may be expected to hinder the T7 RNA polymerase from transcribing mRNA from pDNAs in a cell-free protein synthesis reaction mixture. In addition, ribosomes work to maintain an exquisite electrostatic balance among themselves. Ribosome-associated proteins are positively charged under physiological conditions,^22^ which is an important feature that helps to orchestrate the sophisticated RNA–protein machinery.^23^ In addition, approximately half of the ribosome surface is coated by ribosomal RNA.^23^ Therefore, it is possible that the strong cationic surface of the 8 mol% DOEPC and 16 mol% DOEPC liposomes induced ribosomal dysfunction and aggregation of the anionic components of the cell-free protein synthesis.

In particular, large polyion complexes like droplets of the liquid–liquid phase separation were formed by the interaction of the ribosomes with the relatively weakly cationic 2 mol% and 4 mol% DOEPC liposomes. Even under the conditions of phase separation, it was interesting that the yield of the cell-free synthesized Cx43 did not change very much. Next, we evaluated the effect of the 2 mol% and 4 mol% DOEPC liposome concentrations on the cell-free Cx43 synthesis (Fig. 1C). For each liposome, the amount of synthesized Cx43 did not significantly vary at the lipid concentration was changed from 0.5 to 4 mM. The average amounts of synthesized Cx43s at each lipid concentration were 99.6–105% for the DOPC liposome, 83.4–91.4% for the 2 mol% DOEPC liposomes and 71.2–80.0% for the 4 mol% DOEPC liposomes compared with the amount of DOPC liposome at 0.5 mM lipid. Therefore, the concentration of cationic liposomes did not affect cell-free membrane protein synthesis so much.

In the case of the 2 mol% DOEPC liposomes, the interaction of the ribosomes as a function of the concentration of the liposomes in 50 mM HEPES buffer was estimated by DLS. As the concentration of liposomes increased, the size of the polyion complex increased (Fig. 1D). When RNase was added to the complexes of ribosomes at various lipid concentrations of 2 mol% DOEPC liposomes, the ribosomes were degraded and the liposome sizes returned to those of the original liposomes. This indicated that the cationic liposomes were isolated from the ribosomes by the treatment with RNase even in the case of a large polyion complex system. Furthermore, using the mixture obtained from the cell-free Cx43 synthesis in the presence of 2 mol% DOEPC liposomes at 4 mM lipid, the nucleic acids components were stained by SYBR Gold, and observed by confocal laser scanning microscopy (Fig. 1E). In the presence of 2 mol% DOEPC liposomes, some submicro- and micro-sized spots were observed compared with the case for the DOPC liposomes where no spots were observed. In addition, the fluorescence intensity profile from a high-magnification image revealed that nucleic acid components were detected at both the edge and inside of a spot (Fig. 1F). This supported the idea that polyion complexes were formed between cationic liposomes and anionic nucleic acid components in the cell-free reaction mixture.

We investigated the chaperoning effect involved in the formation of the Cx43-reconstituted liposomes after the cell-free protein synthesis in the presence of 2 mol% DOEPC liposomes. After the ultracentrifugation of the cell-free reactants (163 000 × *g* at 4 °C for 2 h), the upper supernatant was collected as the liposome fraction and the lower sediment was collected as the aggregation fraction (Fig. 2A). In the presence of 0.5 mM lipid, the percent of Cx43 in the liposome fraction compared with the total amount was (51.6 ± 4.4) % and (26.7 ± 2.8) % for DOPC and 2 mol% DOEPC, respectively. In the presence of 4 mM lipid, the percent increased to (74.2 ± 5.1) % and (76.4 ± 3.4) % for DOPC and 2 mol% DOEPC, respectively.

**Fig. 2:**
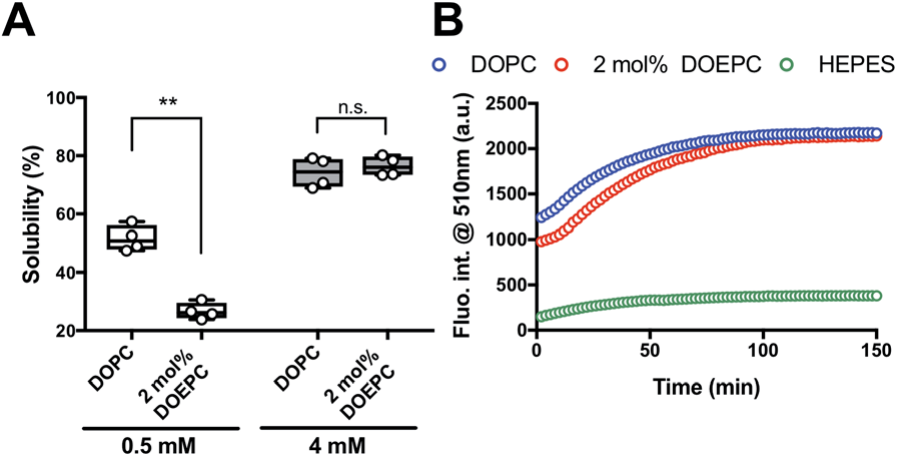
Investigation of EGFP folding as function of connexin-43 solubilization. (A) Solubility of Cx43 in the presence of liposomes semi-quantified after ultracentrifugation. Each dot denotes an individual solubility measurement (*n* = 4). The line denotes the mean. (B) Time-course of fluorescence intensity at 510 nm at 37 °C, which corresponds to the folded Cx43-EGFP produced by cell-free protein synthesis.

To evaluate whether Cx43 was correctly folded using this system, a Cx43–enhanced green fluorescent protein (EGFP) fusion protein was employed because previously, an EGFP fusion constructed at the C-terminus of a membrane protein was demonstrated to be useful as a folding indicator in cell-free membrane protein synthesis.^24^ The time-course of the fluorescence intensity of cell-free synthesized Cx43–EGFP was measured in the presence of DOPC liposomes and 2 mol% DOEPC cationic liposomes (Fig. 2B). In the absence of the liposomes, the fluorescence intensity of Cx43–EGFP was hardly detected at any time points probably because of the aggregation of Cx43–EGFP (Fig. S1, ESI†). In contrast, the fluorescence intensity of Cx43–EGFP increased as it was correctly folded in the presence of both DOPC and 2 mol% DOEPC liposomes. Both fluorescence intensities reached a plateau at 100 min and were similar (Fig. 2B). The total amount of folded Cx43–EGFP inserted into the liposomal membrane was almost comparable for the 2 mol% DOEPC cationic liposomes and DOPC liposomes. Most Cx43s in cationic and nonionic proteoliposomes would likely be correctly folded by the chaperoning effect of these liposomes during cell-free protein synthesis.

In a previous study, the formation of the bioactive hemichannel pore of Cx43 was evaluated by the release of the fluorescent probe, ANTS (8-aminonaphthalene 1,3,6-trisulfonic acid) from the inside of the liposome to the outside.^12^ The release experiment was carried out by using 2 mol% DOEPC proteoliposomes containing ANTS during cell-free Cx43 membrane protein synthesis (Fig. 3). The fluorescence of ANTS released from the inside of the liposomes was quenched by the addition of 120 mM *p*-xylene-bis-pyridinium bromide (DPX) to the reactants, and the fluorescent intensity of residual ANTS was measured in the liposome. In the control experiments, ANTS remained inside both the DOPC liposomes and 2 mol% DOEPC cationic liposomes even in the presence of reactants from the cell-free protein synthesis. However, the amounts of ANTS in the CX43 proteoliposome systems were less than that of the control system. This result indicated a portion of the ANTS leaked outside of the liposomes because of the formation of the pore by the Cx43 hemichannel during the cell-free membrane protein synthesis. Over 80% of ANTS was released from both the DOPC liposomes and 2 mol% DOEPC cationic liposomes. The similar release behavior of the nonionic and cationic proteoliposome systems was comparable with the results of the solubility and the folding experiments. The results suggested that cell-free synthesized Cx43 was incorporated into the cationic liposomal membrane and formed the bioactive hemichannel.

**Fig. 3:**
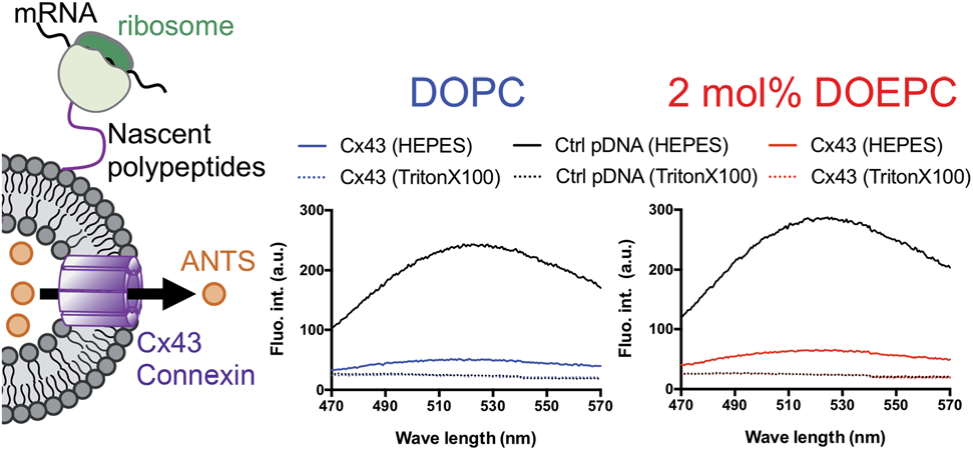
Permeation of a fluorescence probe through the connexon. Cell-free membrane protein synthesis using pURE-Cx43 (colored line), and pURE1 (black line) was performed in the presence of liposomes. Fluorescence spectroscopy of ANTS after addition of DPX.

In summary, the effect of DOEPC/DOPC cationic liposomes on cell-free membrane protein synthesis using the channel-forming membrane protein Cx43 was reported. Cationic liposomes containing a higher amount of DOEPC cationic lipid inhibited cell-free protein synthesis because of interactions with anionic components such as ribosomes, DNA and RNA. In particular, cationic liposomes containing a lower amount of DOEPC (2 mol%) interacted with ribosomes and formed a large aggregate-like polyion complex, however, this aggregation did not significantly inhibit cell-free protein synthesis (Scheme 1). Additionally, the cationic liposomes showed a chaperoning effect on both the reconstitution of Cx43 to the cationic liposome and the formation of the higher order structure of the active hemichannel. We envisage the use of the cationic proteoliposomes as a tool for cell biology and, especially, DDS nano carrier research field. Various functional membrane proteins can be reconstituted and functional cationic proteoliposomes prepared by a cell-free membrane protein synthesis/cationic liposome system. Thus, this preparation method could open up the advanced application for the membrane protein-conducted DDS.

**Scheme 1:**
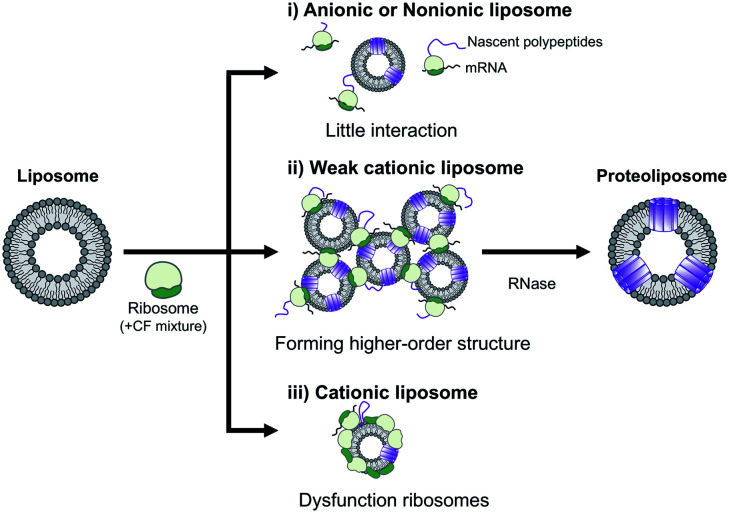
Schematic illustration of the electrostatic interactions between liposomes and ribosomes in a cell-free protein synthesis reaction mixture.

## Conflicts of interest

There are no conflicts to declare.

## Supplementary Material

RA-010-D0RA05825D-s001
