# Supplementary material for: Preparation of cationic proteoliposomes using cell-free membrane protein synthesis: the chaperoning effect of cationic liposomes
Source: RSC Adv. 2020 Aug 4;10(48):28741–5. doi: 10.1039/d0ra05825d (PMC9055869; doi:10.1039/d0ra05825d)
Supplement: RA-010-D0RA05825D-s001 [file RA-010-D0RA05825D-s001.pdf]

Electronic Supplementary Material (ESI) for RSC Advances.
This journal is © The Royal Society of Chemistry 2020

Electronic Supplemental Information (ESI)
for

Preparation of Cationic Proteoliposomes using Cell-free Membrane Protein Synthesis: The Chaperoning Effect of Cationic Liposomes

Mitsuru Ando, Yoshihiro Sasaki and Kazunari Akiyoshi*

Department of Polymer Chemistry, Graduate School of Engineering, Kyoto University, Katsura, Nishikyo-ku, Kyoto, 615-8510, Japan.

*: corresponding author

Materials and Methods

Plasmid DNA preparation

The rat Cx43-expressing plasmid DNAs (pDNA), pURE-Cx43 (denoted as pURE2-Cx43) and pURE-Cx43-EGFP (denoted as pURE2-Cx43-EGFP) were prepared as described previously.[1] The pDNA was amplified in E. coli DH5α. The pDNA was purified using a PureLink® HiPure Plasmid Midiprep Kit (Thermo Fisher Scientific, Waltham, MA, USA).

Liposome preparation

1,2-Dioleoyl-sn-glycero-3-phosphatidylcholine (DOPC) was purchased from Nichiyu (Tokyo, Japan). 1,2-Dioleoyl-sn-glycero-3-ethylphosphocholine (chloride salt) (DOEPC) was purchased from Avanti Polar Lipids (Alabaster, AL, USA). The liposomes were obtained using the following lipid mixtures: DOPC and [DOPC/DOEPC (84:16, 92:8, 96:4, 98:2 and 99:1) mol% ratio]. Liposomes were prepared using a conventional extrusion method as described previously.[2] In brief, the lipid film was hydrated with 50 mM HEPES buffer (pH 7.2), and incubated for 2 h at 37 °C. Following hydration, the

liposome suspensions were extruded with a mini-extruder (Avanti Polar Lipids) equipped with a 0.1-μm pore polycarbonate membrane (Whatman, Maidstone, UK) at 50 °C. The average size and zeta (ζ) potential of the liposomes were measured using a Zetasizer Nano ZSP instrument (Malvern Instrument Inc., Worcestershire, UK). The lipid concentration was measured using the Phospholipid C-Test from Wako (Osaka, Japan).

Measurement of the interactions between cationic liposomes and ribosomes

The average size of liposomes and ribosomes were measured as described above. Cationic liposomes containing 0.5 mM lipid were incubated with a 2-μM ribosome solution (GeneFrontier) in 50 mM HEPES buffer (pH 7.2) for 30 min at 37 °C. After incubation, ribosomes were destructed by adding RNaseOne (Promega, Madison, WI, USA) to the solution.

Preparation and purification of membrane protein-integrated proteoliposomes

Membrane protein-integrated liposomes were prepared using PURESYSTEM (PUREfrex®1.0; GeneFrontier, Chiba, Japan) as described previously.[2] In brief, the

reaction mixture containing 4 ng/μL pDNA for Cx43 or 3 ng/μL pDNA for Cx43-EGFP was incubated with or without the indicated concentration of lipids (liposomes) without agitation for 4 h at 37 °C in a heat block incubator. For purification, a 40 μL sample was overlaid with 40 μL of 15% (w/v) sucrose solution and ultracentrifuged at 163,000 × g at 4 °C for 2 h. The 60 μL upper layer was collected as the supernatant sample and the lower 20 μL fraction was collected as the pellet sample.

Evaluation of the amount of synthesized connexin-43 and its solubility

To evaluate the amount of synthesized Cx43 and Cx43-EGFP, western blot analysis was performed using a SNAP i.d. ® 2.0 Protein Detection System (Millipore, Billerica, MA, USA) as described previously.[2] In brief, after separation by SDS-PAGE, protein bands were transferred electrophoretically to a polyvinylidene difluoride membrane. After blocking with Blockingone (Nacali Tesque, Kyoto, Japan), the membrane was incubated with a mouse anti-connexin-43 monoclonal IgG (BD Transduction Laboratories™, Lexington, KY, USA), and then incubated with goat anti-mouse IgG conjugated with horseradish peroxidase (Santa Cruz Biotechnology, Santa

Cruz, CA, USA). Then the membrane was reacted with ECL Western Blotting Detection Reagent (GE Healthcare) and bands were visualized and quantified using ImageQuants™ LAS4000 (GE Healthcare).

Confocal laser scanning microscopy (CLSM) observations

After the cell-free Cx43 synthesis in the presence of 4 mM lipid (liposomes), the cell-free reactants were added to a solution of SYBR Gold (1:2,500 dilution; Molecular Probes™/Thermo Fisher Scientific, Eugene, OR, USA). After mixing gently, the image was captured using CLSM (LSM780; Carl Zeiss, Oberkochen, Germany) with an excitation wavelength of 488 nm for SYBR Gold.

Measurement of connexin-43-EGFP production

The fluorescence intensity of Cx43-EGFP was measured at an excitation wavelength of 480 nm and emission wavelength of 510 nm using a fluorescence spectrometer (FP-8500; JASCO, Tokyo, Japan) equipped with Peltier thermostat cell holder system (ETC-815; JASCO). The cell-free reaction mixture containing 3 ng/μL

pURE-Cx43-EGFP was incubated with or without 4 mM lipid (liposomes) at 37 °C and the time-course of synthesized cell-free Cx43-EGFP was recorded by measuring the increase in fluorescence intensity.

Dye transfer assay

8-Aminonaphthalene-1,3,6-trisulfonic acid, disodium salt (ANTS) (Molecular Probes™/Thermo Fisher Scientific, Eugene, OR, USA) containing liposomes was prepared as described previously[2]. The cell-free Cx43s protein synthesis was performed for 4 h in the presence of liposomes (0.5 mM lipid). Immediately after the cell-free Cx43s synthesis, the reactants were mixed with an equal volume of 50 mM HEPES buffer (pH 7.2) with or without 1% Triton X-100 and an equal volume of 50 mM HEPES buffer containing 360 mM p-Xylene-Bis-Pyridinium Bromide (DPX) (Molecular Probes™/Thermo Fisher Scientific). Fluorescence intensities were measured with a fluorescence spectrometer (JASCO) at an excitation wavelength of 350 nm and emission wavelength of 530 nm.

Statistical analysis

Differences in the solubility of Cx43 using the liposomes containing difference ratios of DOEPC and the inhibitory effects of the DOEPC liposomes on cell-free Cx43 synthesis were statistically evaluated using one-way factorial ANOVA followed by Tukey's multiple comparisons test. An adjusted $P < 0.05$ was considered statistically significant. All statistical analyses were performed using Graphpad Prism 7 (GraphPad Software, Inc., La Jolla, CA, USA).

References

[1] Y. Moritani, S. M. Nomura, I. Morita, K. Akiyoshi, *The FEBS journal* **2010**, 277, 3343.

[2] M. Ando, S. Schikula, Y. Sasaki, K. Akiyoshi, *Adv Sci (Weinh)* **2018**, 5, 1800524.

Table S1. Size and zeta potential of liposomes in 50 mM HEPES buffer (pH 7.2).

	Size (d.nm)	PdI	Zeta-potential (mV)
DOPC	135.3 ±2.0	0.075	0.72 ± 0.2
1 mol% DOEPC/DOPC	145.0 ± 2.0	0.166	7.19 ± 0.7
2 mol% DOEPC/DOPC	127.1 ± 3.0	0.110	11.8 ± 1.2
4 mol% DOEPC/DOPC	132.0 ± 2.1	0.155	25.6 ± 1.8
8 mol% DOEPC/DOPC	127.2 ± 0.8	0.139	34.6 ± 5.7
16 mol% DOEPC/DOPC	125.2 ± 1.3	0.117	65.4 ± 5.9

Results are expressed as the mean ± standard deviation (n = 3).

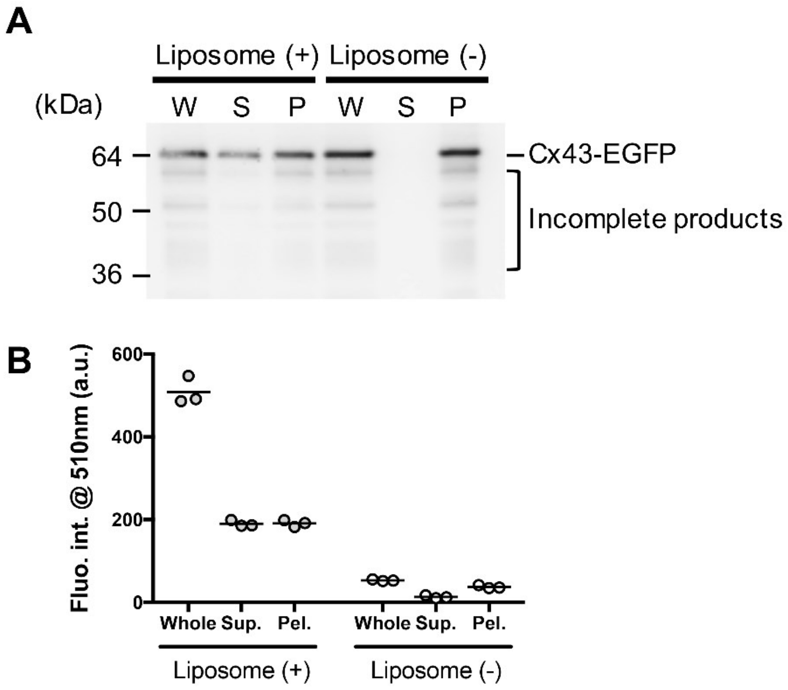

Figure S1. The solubility and the correct folding of cell-free synthesized connexin43-EGFP. (A) Solubility of Cx43-EGFP in the presence of DOPC liposomes (4 mM lipid) was confirmed by western blot analysis after ultracentrifugation. The cell-free whole reactant (W) was separated by ultracentrifugation, resulting in supernatant (S) and pellet (P). (B) The fluorescence intensity at 510 nm, which corresponds to the production of folded Cx43-EGFP was measured.
